# The diagnostic yield, candidate genes, and pitfalls for a genetic study of intellectual disability in 118 middle eastern families

**DOI:** 10.1038/s41598-022-22036-z

**Published:** 2022-11-07

**Authors:** Ghalia Al-Kasbi, Fathiya Al-Murshedi, Adila Al-Kindi, Nadia Al-Hashimi, Khalid Al-Thihli, Abeer Al-Saegh, Amna Al-Futaisi, Watfa Al-Mamari, Abdullah Al-Asmi, Zandre Bruwer, Khalsa Al-Kharusi, Samiya Al-Rashdi, Fahad Zadjali, Said Al-Yahyaee, Almundher Al-Maawali

**Affiliations:** 1 Department of Genetics, College of Medicine and Health Sciences, Sultan Qaboos University, Muscat, Oman; 2 Genetic and Developmental Medicine Clinic, Sultan Qaboos University Hospital, Muscat, Oman; 3 Department of Pediatrics, Royal Hospital, Ministry of Health, Muscat, Oman; 4 Department of Child Health, College of Medicine and Health Sciences, Sultan Qaboos University, Muscat, Oman; 5 Department of Medicine, College of Medicine and Health Sciences, Sultan Qaboos University, Muscat, Oman; 6 Department of Biochemistry, College of Medicine and Health Sciences, Sultan Qaboos University, Muscat, Oman

**Keywords:** Diseases, Neurology, Genetics, Clinical genetics, Consanguinity, Medical genetics

## Abstract

Global Developmental Delay/Intellectual disability (ID) is the term used to describe various disorders caused by abnormal brain development and characterized by impairments in cognition, communication, behavior, or motor skills. In the past few years, whole-exome sequencing (WES) has been proven to be a powerful, robust, and scalable approach for candidate gene discoveries in consanguineous populations. In this study, we recruited 215 patients affected with ID from 118 Middle Eastern families. Whole-exome sequencing was completed for 188 individuals. The average age at which WES was completed was 8.5 years. Pathogenic or likely pathogenic variants were detected in 32/118 families (27%). Variants of uncertain significance were seen in 33/118 families (28%). The candidate genes with a possible association with ID were detected in 32/118 (27%) with a total number of 64 affected individuals. These genes are novel, were previously reported in a single family, or cause strikingly different phenotypes with a different mode of inheritance. These genes included: *AATK, AP1G2, CAMSAP1, CCDC9B, CNTROB, DNAH14, DNAJB4, DRG1, DTNBP1, EDRF1, EEF1D, EXOC8, EXOSC4, FARSB, FBXO22, FILIP1, INPP4A, P2RX7, PRDM13, PTRHD1, SCN10A, SCYL2, SMG8, SUPV3L1, TACC2, THUMPD1, XPR1, ZFYVE28.* During the 5 years of the study and through gene matching databases, several of these genes have now been confirmed as causative of ID. In conclusion, understanding the causes of ID will help understand biological mechanisms, provide precise counseling for affected families, and aid in primary prevention.

## Introduction

Global Developmental Delay (GDD)/Intellectual disability (ID) represents a group of genetic, phenotypic, and clinically heterogenic disorders that affect approximately 1% of children worldwide^1^. Significant limitations define GDD or ID in both intellectual functioning and adaptive behavior that originates during brain development. Non-genetic causes such as infections, autoimmunity, and environmental factors are described, but the majority of such disorders have a genetic basis^2^.

Hundreds of genes are thought to be involved in the etiology of ID^3^. The list of ID genes has expanded and according to the SysNDD database, there are now 2841 primary and candidate human ID genes^4^. In the last decade, advances in genetic technologies such as next-generation sequencing (NGS) have revolutionized clinical practice in medical genetics, aided clinical diagnosis, and proved to be very effective in discovering an ever-increasing number of ID-related genes. They have also enabled deciphering the ID’s heterogeneous genetic mechanisms^5^.

Whole-Exome Sequencing (WES), as a clinical diagnostic test, has a success rate of about 30–40%^6^. The diagnostic yield of chromosomal microarray in children with no underlying cause of their ID is around 15 to 20%^7^. In trio-based WES done in groups of children with severe ID, the yield ranged from 13 to 35%^8^. In contrast, exome sequencing in samples from consanguineous families with various ID-associated phenotypes has produced a high yield. For example, in several studies of ID from the Middle East, NGS yield ranged from 37 to 90%, depending on the patients’ cohorts, the study design, and the classification of the variants^9–15^.

This study reports on the diagnostic yield and candidate genes of a genetic study of intellectual disability in 118 Omani families. The study period extended over five years and included 215 affected individuals. The diagnostic yield when considering both pathogenic and uncertain variants was 55%. Candidate genes with a possible association with ID phenotype seen in this cohort were detected in 32/118 (27%) with a total number of 64 affected individuals. Understanding ID causes will provide precise counseling for affected families and aid in primary prevention. Also, the costs of unnecessary investigations will be spared, with fewer diagnostic odysseys. The paper also discusses the pitfalls and challenges of candidate gene discovery in a consanguineous population.

## Materials and methods

### Human subjects

The Medical Research Ethics Committee approved the study of Sultan Qaboos University (SQU MREC#1362). Informed written consent was obtained from all participants or their guardians. All methods were performed in accordance with the relevant guidelines and regulations and in accordance with the declaration of Helsinki. The target patients included in this study presented with global developmental delay or intellectual disability, all assessed clinically by medical geneticists (detailed methods in SUPP_S1). Severe phenotypes causing death within the neonatal or early infantile period were also included as a neurological phenotype was evident, such as seizures, hypotonia, or brain malformations. Families with a likely autosomal recessive pattern of inheritance were selected. Patients with known molecular diagnoses at the time of recruitment were excluded. The study and exome data analysis were carried out over 5 years, between 2016 and 2021.

### Whole exome sequencing and variant interpretation

Whole-exome sequencing analysis was performed for all affected individuals where samples were available. A detailed methodology is presented in the supplemental data (SUPP_S1). In brief, the method used hybrid capture technology (Agilent SureSelect Human All-exons-V6 or V7) for exome enrichment and capture. Illumina technology (Hiseq2500, Hiseq4000, or NovaSeq6000) of 150 bp paired-end, at 150-200X coverage, was used for sequencing. The reads were mapped against UCSC GRCh37/hg19 or GRCh38/hg38. Filtering and variant prioritization were analyzed using an in-house pipeline (SUPP_S1). Variant filtration was performed to keep only novel or rare variants (≤ 1%). Public databases such as 1000 Genomes, Exome Variant Server, and GnomAD were used for alleles frequencies. For filtration of common variants against the Middle Eastern population, the Greater Middle East (GME) and variome database “al mena” that comprises data of 2497 samples was used^16,17^. Our in-house population-specific exomes database, which contains data of 1564 WES, was also used. During the filtration process, the phenotype and mode of inheritance were both considered. Any potential variants identified after prioritization were further confirmed by Sanger sequencing. Members included in the segregation analysis ranged from 3 to 12 members of each family, depending on the DNA availability. Most of the segregation was performed for the parents and siblings alongside the index patient. When a definitive cause was not possible or when a candidate gene was considered, further analysis of copy number variants was performed on exome data using ExomeDepth^18^ (SUPP_S1).

Classification of variants was based on the published ACMG guideline^19,20^. Pathogenic or likely pathogenic variants in known disease-causing genes which could be linked to the reported phenotypes of the affected patients were categorized as disease-causing variants. The second category was for the variants in known disease-causing genes that overlapped with the patient’s phenotype, and these were considered possible disease-causing variants. These were rare and damaging variants of uncertain significance (VUS). Variants in candidate genes, which were predicted to be deleterious and found in genes not previously confirmed to be implicated in human disease, formed the third category. These genes are novel; they were previously reported in a single family or cause strikingly different phenotypes with different modes of inheritance. Supporting data for candidate genes included variants within a shared autozygosity area. The variant is of high or moderate impact; the population frequency supports genic intolerance to such variants, and in-silico prediction tools indicate a damaging effect. Data for gene function and network, gene expression, and animal models were also considered.

## Results

Whole-exome sequence analysis was performed for 188 individuals representing 118 characterized families with a total number of 215 affected individuals. Of the 118 families included, 93 (78.8%) had a family history of one or more affected individuals in addition to the index patient, all with a similar phenotype. The age range of the affected individuals, at first clinical assessment, was from birth to 34 years old. The average age at which WES was completed was 8.5 years, and children below five years of age, represented 30% of the affected individuals when WES was completed. Males represented 57.2% of the studied group (123 Males:92 Females). The rate of consanguineous marriages within the included families was 91%. The affected patients exhibited diverse phenotypes, including global developmental delay, seizures, brain malformations, microcephaly, facial dysmorphism, and other systemic manifestations (Table 1 and Supplementary SUPP_Table1).

A total of 420 members’ DNA samples were available for WES or Sanger sequencing for segregation analysis. These included healthy or affected members. However, DNA samples were not available for analysis in 22 out of the 215 affected individuals. Sanger sequencing was used to confirm the variant and phenotype-genotype segregation in all candidate variants. Only variants that were confirmed and segregated with the phenotype are reported.

Variants in previously known and described ID genes were seen in 65/118 families (55%). Following the ACMG guidelines of variant classification, pathogenic (P) or likely pathogenic (LP) variants were detected in 32/118 families (27%). Variants of uncertain significance were seen in 33/118 families (28%). The majority of these two groups showed homozygous variants (51/65; 78.5%). These variants are rare; they explain the disease manifestations, are predicted to be damaging, are confirmed by Sanger sequencing, and are segregated with the phenotype (All listed in SUPP_Table1).

**Figure 1:**
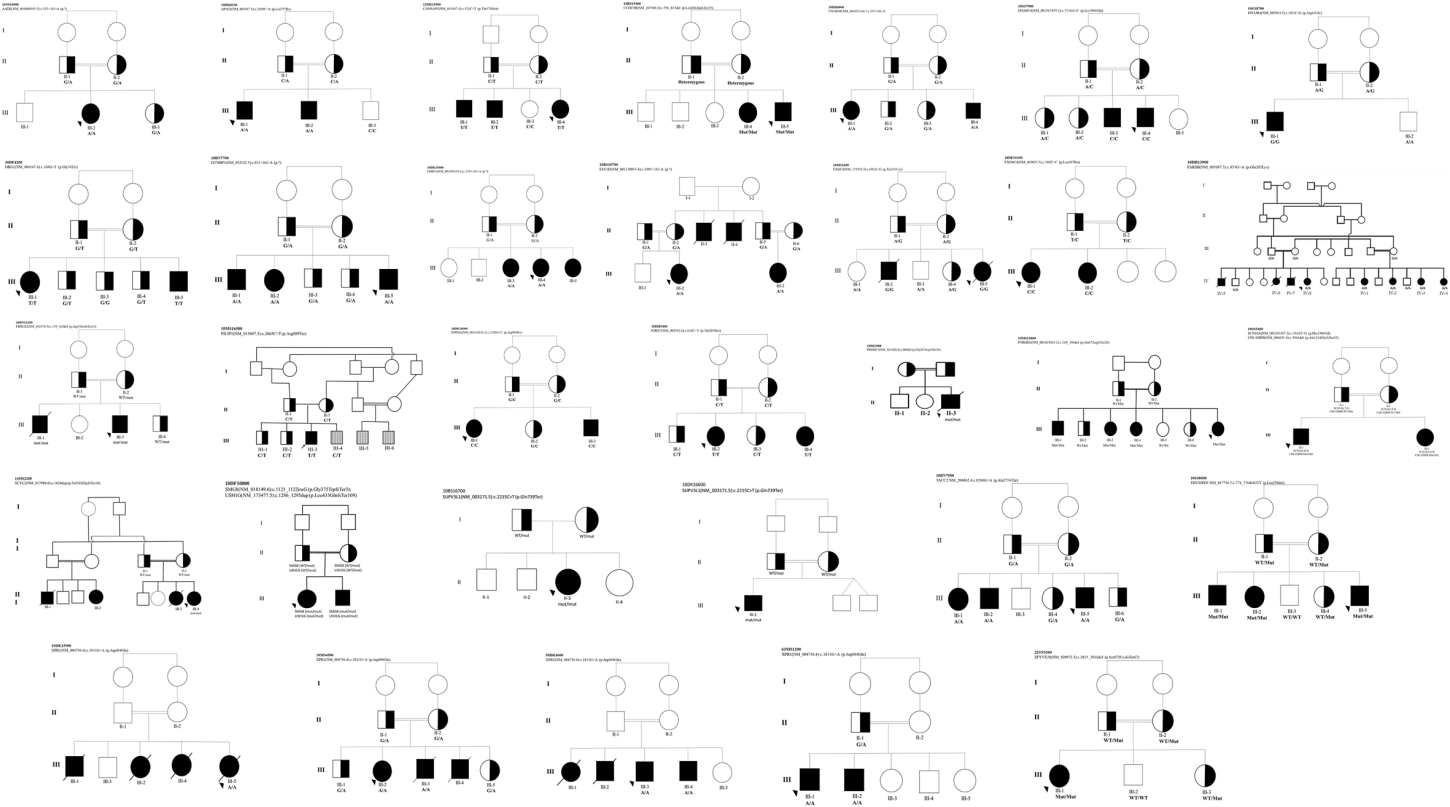
Pedigrees of families with candidate genes, showing the variants. Shaded symbols indicate affected individuals and arrows indicate the proband, zygosity for the variant is included.

Candidate genes with a possible association with ID phenotype seen in this cohort were detected in 32/118 (27%) with a total number of affected individuals of 64 (Table 1). These candidate genes were selected according to rarity and absence in homozygosity status in local control exomes or public databases. The impact of the variants is predicated damaging. The expression patterns or mouse models supported an association with neurological dysfunction. Importantly, Sanger sequencing confirmed segregation for all variants in candidate genes in up to 3 generations in the family pedigrees (Fig. 1). The total number of candidate genes identified was 28. Table 1 shows detailed findings for the candidate genes.

Twenty-one families (18%) with 40 affected individuals remain unsolved despite multiple molecular tests, including WES, chromosomal microarray, and Fragile X where applicable. There is a trend of milder phenotypes and usually non-syndromic intellectual disabilities in unresolved cases. However, we can not draw a firm conclusion because of the small numbers.

## Discussion

Global Developmental Delay (GDD)/Intellectual disability (ID) represents a group of genetic, phenotypic, and clinically heterogenic disorders that affect approximately 1% of children worldwide. This study presents the results of 188 exome analyses representing 118 consanguineous Omani families. This cohort included 215 affected individuals with intellectual disabilities, including global developmental delay, seizures, brain malformations, microcephaly, facial dysmorphism, and other systemic manifestations. Overall 82% were found to have a possible explanation. Specifically, 55% had variants in previously described and known genes (P/LP or VUS) and 27% in possible candidate genes.

With the enrichment for consanguineous families (91%), it was not surprising that the majority (85.5%) of the overall variants in the three groups were homozygous. The consanguinity rate is high due to the preference to include families with autosomal recessive phenotypes and multiple affected individuals. This study detected pathogenic (P) or likely pathogenic (LP) variants in 32 families, making the diagnostic rate of the study 27%. The main goal of this study was to recruit unsolved cases. However, the diagnostic rate observed was higher than anticipated. This can be explained by frequent reanalysis of exome data during the last five years of the study, thus enabling newly published genes to be detected. Also, VUSs, including non-coding variants, were selected with further evidence of pathogenicity becoming available. Also, during the study's initial phase, some families did not have access to clinical exome sequencing and thus were channeled to the research exome.

In a large-scale exome sequencing study, Monies and colleagues^15^ reported the yield of exome sequencing on 2219 families from Saudi Arabia. The overall diagnostic yield of exome sequencing based on cases with confirmed pathogenic or likely pathogenic variants was 43.3%. However, if considering variants of unknown significance (VUS) that are in an established disease-related gene or candidate genes with compelling biological candidacy were considered, the yield rate would be 73%. The high throughput design of this study led to the discovery of 236 genes that have no established OMIM phenotypes and were proposed as candidate genes. The negative results (unsolved cases) accounted for 27% of the total.

The total number of candidate genes for intellectual disability identified in this study was 28. During the course of this study, Gene Matcher^21^ was used to provide further evidence of association with the phenotype. Through this study and in collaboration with the scientifc community, several of these genes have been successfully confirmed to cause intellectual disability (Table 1). One interesting shared candidate variant within the *XPR1* gene was identified in multiple affected individuals from four apparently unrelated families (Table 1). These families come from different geographical areas of Oman. However, haplotype analysis using exome data indicated that they all shared the same haplotype (Data not shown). The XPR1 protein functions to mediate phosphate export from the cell as well as binding inositol hexakisphosphate and related inositol polyphosphates, which are key intracellular signaling molecules^22^. Mutations in the *XPR1* gene are known to be associated with the dominant condition of idiopathic basal ganglia calcification-6; OMIM 616413^23^. The earliest age of onset for this condition is in the third and fourth decade of life, with symptoms of cerebrovascular insufficiency associated with movement disorders, cognitive decline and psychiatric symptoms. Our patients’ phenotype is strikingly different; we detected biallelic *XPR1* variants and apparently healthy parents. The phenotype included variable signs of neonatal pulmonary hypertension, cardiomyopathy, serum hypophosphatemia, chronic lung disease requiring oxygen therapy, severe developmental delay, and brain basal ganglia calcification. Further functional characterization for these variants has commenced.

Whole-genome sequencing (WGS) analysis can cover up to 98% of the human genome, whereas WES only covers about 95% of the coding regions and only 1–2% of the genome. WES has a lower cost per sample than WGS, a greater depth of coverage in target regions, lesser storage requirements, and easier data analysis^24^. It is, however, worth highlighting the pitfalls and challenges that can occur when WES analysis is performed. WES is a high-throughput, complex technique with potential pitfalls at every step. These pitfalls and the consequential missing of the molecular diagnosis in exome sequencing and analysis is a recognized phenomenon^25^. This phenomenon could be caused by various factors, including technological limitations in variant detection, a lack of enrolling additional family members, many variants identified for probands, or the causal variant being located outside of the coding regions^25^. Pitfalls related to WES analysis can be categorized into three main groups (Table 2).

The first group of pitfalls consists of those which are sequence-related. Large rearrangements or complex structural variants are one example. Structural variants are genomic rearrangements larger than 50 bp in size, and they account for about 1% of the variation in human genomes^26^. Complex structural variants have been shown to contribute to human genomic variation and to cause Mendelian disease^27^. Unfortunately, these cannot be identified easily by WES. However, multiple pipelines for CNV analysis are available. In this study and using ExomeDepth, it was possible to detect CNVs in a homozygosity state as a cause of the phenotype (Families 11MS8800 and 10DH12500). Mitochondrial mutations are other causative factors that WES cannot detect. Other factors also include mosaicism, abnormal methylation, and uniparental disomy.

Other causes of missing variants in WES include decreased coverage, locus-specific features such as GC-rich regions, and sequencing biases^28,29^. Difficulty in the alignment of indels (insertions/deletions) larger than 20–50 nucleotides long is one of the limitations, which is likely to be the reason for WES missing such variants^30^. Incomplete human genome annotation and sequence might also affect the accuracy of variant mapping and annotation. For instance, an intronic variant could be located in an unannotated exon^31^. Another potential cause is the high sequence similarity between pseudogenes and their corresponding functional genes^32^.

The second group to consider is pitfalls due to annotation and prioritization errors. Annotation and prioritization steps are used in WES analyses to reduce thousands of variants to a few candidates. During the filtering process, all annotations except for the “canonical” transcripts (i.e., the longest transcript of a gene) are initially ignored. Remarkably, pathogenic variants can be missed if alternative transcripts are not fully considered^33^. Splicing is thought to be involved in 15–30% of all inherited disease variants^34^. Despite advances in exome capture methods or machine learning for detecting variants that affect splicing, accurate detections of deep intronic variants remain limited and this could be the reason for missing splicing variants. In our cohort, intronic variants that were likely to affect splicing were detected in 19/118 families (16.1%) of which 8 were in non-canonical splicing sites.

Databases like OMIM and HGMD are used to find gene-disease and variant–disease associations in the literature. Variants or genes listed in these databases would be flagged as potentially disease-causing^35^. Nevertheless, a reason for an initial false-negative result is that such variants or disease databases have not been kept up to date. A recent study by Bruel and colleagues illustrated this issue in a study cohort of 313 individuals^36^. Likewise, when considering deleterious variants, those with high population prevalence might be filtered out. Penetrance of the disease might be influenced by numerous factors, including other hidden rare variants, family history, inheritance, additional medical problems, and ethnic background.

Synonymous variants, which are known as ‘silent’ variants, represent almost 50% of the variant list identified by WES. Filtering out synonymous variants reduces the variants’ list because they are assumed to be benign. However, our increased knowledge about the relationship between genetic variants and disease has shown that synonymous variants play a significant role in human disease risk and other complex traits, including variants that affect splicing^37^. Indeed, a recent study challenged the concept that synonymous variants are neutral. In their yeast study, Shen and colleagues showed a strong non-neutrality of most synonymous mutations^38^. If this holds true for other genes and organisms, then numerous biological conclusions, including disease causation about synonymous mutation, would require re-examination. An example in our cohort is the family 16SS2600, where GPT2 (p.Gly245Gly) was initially missed and flagged as a silent variant.

All labs encounter pitfalls related to clinical factors and phenotypes. As a result, a negative WES result must be interpreted in the context of the patient’s clinical history to determine whether reevaluation or further testing is necessary. For example, within our cohort (family 10MS6600), two related families with multiple affected individuals were enrolled as having the same phenotype and WES analysis was initially negative. However, after a detailed clinical reevaluation and exome reanalysis, the results showed that two different diseases were possibly running in the family. Some of the affected members were indeed found to harbor a deep intronic variant in the *PGAP3*, which was recently reported to cause hyperphosphatasia with mental retardation syndrome type 4 (OMIM 615,716). Another example is family 10MS16500, demonstrating that multiple individuals carrying two or more different diseases can complicate the phenotype. Similarly, the family (10DF10800) with multiple affected individuals presented with developmental delay, congenital cataracts, and bilateral sensorineural hearing loss. WES analysis identified two different variants for two different syndromes in two genes, one of which is novel as the underlying cause^39^. Another aspect is the mode of inheritance in which conditions are known to be autosomal dominant but manifest as autosomal recessive. Monies and colleagues reported many examples of genes or diseases inherited as both AD and AR^15^.

**Table 1:** Candidate Genes. Several were published previously through gene matching.

Family-ID	Index's Age at exome test	Index's Gender	Other Affected	Phenotype	Gene-Approved	HGVS variant (hg19)	Zygosity	Consanguinity	avsnp150 rs	GnomAD frequency	Pathogenicity Scores *	Function & experimental models	Remarks
11SS10900	2 Years	Female	No	Refractory seizures and developmental delay	AATK	AATK(NM_001080395.3):c.533 + 1G > A (p.?)	homozygous	Yes	NA	Absent	NA	Apoptosis-associated tyrosine kinase (AATK) is a non-receptor type tyrosine kinase whose expression is up-regulated during the apoptosis and differentiation of 32Dcl3 myeloblastic cells. AATK plays an essential role in mature neurons and neuronal differentiation^40,41^	No published genotype–phenotype association
10DK8100	12 Years	Male	Yes	Autistic features, intellectual disability, hyperactivity, and normal MRI brain	AP1G2	AP1G2(NM_003917.5):c.2269C > A (p.Leu757Ile)	homozygous	Yes	rs764435650	0.0000319	0/4	The AP1G2 gene encodes gamma-2-adaptin, a protein very similar to gamma-adaptin (AP1G1), a subunit of the AP1 adaptor complex that engages clathrin to the trans-Golgi network. It might have a role in protein sorting in late endosomes or multivesicular bodies^42^. Zebrafish whole-mount in situ hybridization confirmed that γ2 adaptin (encoded by AP1G2) is detected in the developing central nervous system (CNS) and knockout in zebrafish embryos showed severe, lethal impairment of CNS development^43^. The partner protein AP1G1 was recently published to cause ID^44^	No published genotype–phenotype association
12DK11500	11 Years	Male	Yes	Facial dysmorphic features, severe neurodevelopmental delay, cortical visual impairment and seizures	CAMSAP1	CAMSAP1(NM_015447.4):c.521C > T (p.Thr174Met)	homozygous	Yes	rs1187560194	0.0000319	1/4	CAMSAP1 is a microtubule-organizing protein that binds to the minus end of non-centrosomal microtubules and regulates their dynamics and organization^45^. It is involved in other processes, including neuron projection development and regulation of cell morphogenesis^46^	Gene Matched (understudy)
10BS15400	1 Year	Male	Yes	Severe intellectual disability, seizures, face dysmorphism, hypopigmented hair and skin	CCDC9B	CCDC9B(NM_207380.2):c.796_815del (p.Leu266AlafsTer35)	homozygous	Yes	NA	Absent	NA	CCDC9B (C15orf52) is expressed in the brain. Mouse KO data indicated a neurological phenotype. It showed decreased exploration in new environments and decreased prepulse inhibition (MGI:2685199)	No published genotype–phenotype association
10DH6800	14 Years	Female	Yes	Short stature, microcephaly, cataract, abnormal teeth, obesity, hypertension, and insulin resistance	CNTROB	CNTROB(NM_001037144.7):c.355 + 1G > A (p.?)	homozygous	Yes	rs752440318	7.95E-06	NA	CNTROB is a centrosomal protein involved in centriole duplication and cytokinesis^47^. It is expressed in the brain. It was observed that inhibiting centriole duplication causes cytokinesis defects. In mice, In hippocampal cells, centrobin formed cytoplasmic dots. It enhanced microtubule formation outside as well as inside the centrosome	Gene Matched (understudy)
10SS7900	7 Years	Male	Yes	Intellectual disability	DNAH14	DNAH14(NM_001367479.1):c.5716A > C (p.Lys1906Gln)	homozygous	Yes	rs868380191	Absent	4/4	DNAH14 encodes a microtubule-associated motor protein that contributes to centrosome integrity. DNAH14 was found to be a causative gene in patients with hydrocephalus. Since DNAH14 encodes axonemal dynein, which is found in motile cilia it is believed to affect cilia physiological function during hydrocephalus pathogenesis^48^. Mutation in DNAH14 was also linked to human embryonic lethality^49^	One study was published recently with no further confirmation^50^, and no associated OMIM phenotype entry
10SN8700	6 Years	Male	Yes	IUGR, recurrent pneumonia (lipoid), ventilator-dependent, inguinal hernia, thin corpus callosum and mild to moderate brain atrophy	DNAJB4	DNAJB4(NM_007034.5):c.181A > G (p.Arg61Gly)	homozygous	Yes	NA	Absent	4/4	DNAJB4 is a member of the highly conserved DNAJ/HSP40 protein family. It functions as a chaperone for E-cadherin, allowing appropriate folding, localization, and stability, implying a post-transcriptional level of control^51^. DNAJB4 binds to the carboxyl tail of the human mu opioid receptor in the CNS^52^. DNAJB4 was found to interact with SDIM1, which is thought to play a particular role in brain cell survival and/or receptor trafficking^53^	No published genotype–phenotype association
30DF4200	12 Years	Female	Yes	Intellectual disability, microcephaly, facial dysmorphism, microphthalmia and eczema	DRG1	DRG1(NM_004147.4):c.160G > T (p.Gly54Ter)	homozygous	Yes	NA	Absent	NA	DRG1 is a microtubule-binding protein that performs various microtubule-related functions^54^. Based on its expression pattern, DRG1 may be involved in neuronal cell proliferation and/or differentiation^55^. DRG1 transcript levels in the developing central nervous system of Xenopus laevis revealed a similar pattern of expression^56^	Gene Matched (understudy)
10BN7700	7 Years	Male	Yes	Normocephalic, global developmental delay, and spastic paraplegia	DTNBP1	DTNBP1(NM_032122.5):c.811 + 6G > A (p.?)	homozygous	Yes	NA	Absent	NA	Dysbindin protein is a significant component of the biogenesis of lysosome-related organelles complex-1 (BLOC-1), which controls protein trafficking in the lysosomal pathway^57^. DTNBP1 has been identified to have a role in the development of schizophrenia by affecting brain development and neurotransmission activity^58^	Studies associated DTNBP1 variants with schizophrenia with no further functional confirmation^59^
10DK15000	12 Years	Female	Yes	Intellectual disability	EDRF1	EDRF1(NM_001202438.2):c.2591-1G > A (p.?)	homozygous	Yes	NA	Absent	NA	EDRF1 is a transcription factor^60^; it is highly expressed in the brain	No published genotype–phenotype association
10BS10700	7 Years	Female	Yes	Global developmental delay, microcephaly, severe visual impairment hypomyelination and hypoplastic corpus callosum	EEF1D	EEF1D(NM_001130053.4):c.1905 + 1G > A (p.?)	homozygous	Yes	rs1364060781	3.99E-06	NA	EEF1D gene encodes three different splice forms proteins in addition to a more recently described isoform called eEF1BδL that functions during cellular stress as a transcription factor for heat shock element-containing genes^61^. Mutations in the EEF1D gene have been associated with neurodevelopmental abnormalities^62^	Gene Matched (understudy)
10MS2400	4 Years	Male	Yes	Severe acquired microcephaly, severe delay, seizures, optic nerve atrophy, and hypotonia	EXOC8	EXOC8(NM_175876.5):c.692A > G (p.Tyr231Cys)	homozygous	Yes	rs1558358137	Absent	4/4	EXOC8 is a subunit of the exocyst complex involved in docking exocytic vesicles with fusion sites on the plasma membrane. It is found to play an essential role in normal cortical development and its perturbation causes complex brain disorders. EXOC8 mutations (one family) are linked to neurodevelopmental disorders with microcephaly, seizures, and brain atrophy^63^	?Neurodevelopmental disorder with microcephaly, seizures, and brain atrophy (MIM# 619,076), Two studies correlated EXOC8 variants with a neurodevelopmental phenotype^63,64^, functional studies of the variant and studies of patient cells were not performed
10DF16100	14 Years	Female	Yes	Prenatal growth restrictions, failure to thrive, short stature, global developmental delay, intracerebral calcification and basal ganglia calcifications, and kidney failure	EXOSC4	EXOSC4(NM_019037.3):c.560 T > C (p.Leu187Pro)	homozygous	Yes	NA	Absent	2/4	EXOSC4 is a non-catalytic component of the RNA exosome complex with 3'- > 5' exoribonuclease activity and involvement in numerous cellular RNA processing and degradation events^65^. EXOSC4 is involved in the nucleic acid metabolic process and positive regulation of cell growth^66^. Mutations in other 3'- > 5' exoribonucleases enzymes were associated with a neurodevelopmental disorder^67,68^	Gene Matched (understudy)
10DH13900	28 Years	Female	Yes	Brain calcification, microcephaly, short stature and lung disease	FARSB	FARSB(NM_005687.5):c.853G > A (p.Glu285Lys)	homozygous	Yes	rs767956337	Absent	1/4	Cytoplasmic phenylalanine-tRNA synthetase is a heterodimer composed of a catalytic alpha subunit, FARSA, and a regulatory beta subunit, FARSB. FARSB is an aminoacyl-tRNA synthetase that charges tRNAs with specific amino acids. It is reported to be associated with neurodevelopmental disorders^69,70^	Published^69^
30BN13200	3 Years	Male	Yes	Symmetrical IUGR, clenched hands, hypertelorism, generalized hypotonia, duodenal atresia and annular pancreas	FBXO22	FBXO22(NM_012170.3):c.159_162del (p.Arg53SerfsTer13)	homozygous	Yes	NA	Absent	NA	FBXO22 is a Member of the F-box protein family, which is characterized by comprising 40-amino acid F-box domain. F-box proteins form complexes with other proteins and act as protein-ubiquitin ligases. FBXO22 plays a critical in carcinogenesis and may participate in the progression of parkinsonism^71,72^	Gene Matched (understudy)
10MS16500	1 Years	Male	Yes	Distal arthrogryposis with contractures of the knees and elbows, congenital clubfoot, muscular hypotonia, and mild learning disability	FILIP1	FILIP1(NM_015687.5):c.2665C > T (p.Arg889Ter)	homozygous	Yes	rs775141616	1.19E-05	NA	FILIP1 regulates the migration of neocortical cells from the ventricular zone by acting via a filamin-A/F-actin axis. It may cause filamin-A degradation^73,74^	Gene Matched (understudy)
10DK16000	8 Months	Female	Yes	Severe Global developmental delay and pontocerebellar hypoplasia	INPP4A	INPP4A(NM_001134224.1):c.2702G > C (p.Arg901Pro)	homozygous	Yes	NA	Absent	4/4	INPP4A catalyzes the hydrolysis of the 4-position phosphate of phosphatidylinositol 3,4-bisphosphate, as well as inositol 1,3,4-trisphosphate and inositol 1,4-bisphosphate^75^. INPP4A-null mice developed neurodegeneration in the striatum and severe involuntary movement disorders (MGI:1,931,123)	One study identified a novel nonsense INPP4A mutation in a family with intellectual disability^76^, with no further functional confirmation and no associated OMIM phenotype entry
10DH9400	11 Years	Female	Yes	Global developmental delay, seizures, and visual problems	P2RX7	P2RX7(NM_002562.6):c.614C > T (p.Thr205Met)	homozygous	Yes	rs140915863	0.000127	0/4	P2RX7 is an ATP receptor that functions as a ligand-gated ion channel. When its native ligand, ATP, is not present, it acts as a scavenger receptor, recognizing and engulfing apoptotic cells (Ivetac et al., 2005). It is responsible for the ATP-dependent lysis of macrophages^77^. It can potentially participate in both rapid synaptic transmission and ATP-mediated lysis of antigen-presenting cells. P2X7R was found to have roles in neuroinflammation and implications in Alzheimer's disease pathogenesis^78^	No published genotype–phenotype association
10DK3900	2 Years	Male	No	Central hypoventilation and apnea, seizures, cerebellar vermis hypoplasia and early death	PRDM13	PRDM13(NM_021620.4):c.800del (p.Gly267AspfsTer34)	homozygous	Yes	NA	Absent	NA	PRDM13 is implicated in transcriptional regulation. It is thought to be a vital component of a highly coordinated transcriptional network that determines the balance of inhibitory versus excitatory neurons in the dorsal spinal cord^79^	Published^80^
10MS13800	11 Years	Female	Yes	Global developmental delay, extrapyramidal features and spasticity	PTRHD1	PTRHD1(NM_001013663.2):c.169_196del (p.Ala57ArgfsTer26)	homozygous	Yes	rs553276736	0.000541	NA	Putative peptidyl-tRNA hydrolase with a PTH2 domain, implying that it works in the ubiquitin–proteasome pathway. It was suggested to be linked to early-onset parkinsonism and cognitive dysfunction^81,82^	Published^83^
10SS5400	4 Years	Male	Yes	Hypotonia, global developmental delay, and seizures. Parents are healthy	SCN10A; CDC42BPB	SCN10A(NM_001293307.2):c.3916 T > G (p.Phe1306Val);CDC42BPB(NM_006035.4):c.3941del (p.Ala1314GlyfsTer35)	homozygous; homozygous	Yes	NA; NA	Absent; Absent	4/4; NA	SCN10A gene encodes the alpha subunit of a voltage-gated sodium channel, an integral membrane glycoprotein responsible for the initial rising phase of action in most excitable cells^84^; CDC42BPB encodes a Serine/threonine-protein kinase that is an important downstream effector of CDC42 and regulates cytoskeleton reorganization and cell migration. Recently loss-of-function variants in this gene were associated with a neurodevelopmental phenotype^85^	SCN10A: one study published as a cause of ID with no further confirmation and no associated OMIM entry^86^, other associated disease is an episodic pain syndrome; CDC42BPB: one study published only describing de novo mutations (autosomal dominate) with no further confirmation^85^. OMIM phenotype: Chilton-Okur-Chung neurodevelopmental syndrome (MIM# 619,841). No biallelic variants for CDC42BPB described
14MS2200	2 Years	Female	Yes	Arthrogryposis, global developmental delay, microcephaly, and optic disc pallor	SCYL2	SCYL2(NM_017988.6):c.1624dup (p.Val542GlyfsTer16)	homozygous	Yes	NA	Absent	NA	SCYL2 regulates the production of excitatory receptors at synapses and plays an essential role in regulating neuronal function, signaling, and brain development^87^. Mutations in this gene have been linked to neurogenic arthrogryposis multiplex congenita-4 with corpus callosum agenesis (MIM# 618,766)	Published^88^
10DF10800	5 Years	Male	Yes	Global developmental delay with severely impaired intellectual function and absent speech, additionally Usher syndrome phenotype	SMG8	SMG8(NM_018149.6):c.1121_1122insG (p.Gly375TrpfsTer3); USH1G(NM_173477.5):c.1286_1295dup (p.Leu433GlnfsTer109)	homozygous	Yes	NA; NA	Absent; Absent	NA; NA	SMG8 gene encodes a component of the cell's nonsense-mediated mRNA decay (NMD) machinery. SMG8 and SMG9 as partners regulate the kinase activity of SMG1	Published^39^
10BS16700	5 Years	Male	No	Severe intellectual disability, spastic quadriparesis, axial hypotonia, abnormal brain MRI, and retinal dystrophy	SUPV3L1	SUPV3L1(NM_003171.5):c.2215C > T (p.Gln739Ter)	homozygous	Yes	NA	Absent	NA	SUPV3L1 encodes a helicase that is localized in the mitochondria. It has been shown in vitro to possess both double-stranded RNA and DNA unwinding activity that is ATP-dependent	Gene Matched (understudy). One study was published recently with no further confirmation^89^
10DK16600	12 Years	Female	No	Intellectual disability, spastic quadriparesis, abnormal brain MRI, neuroregression, seizures, and optic neuropathy	SUPV3L1	SUPV3L1(NM_003171.5):c.2215C > T (p.Gln739Ter)	homozygous	Yes	NA	Absent	NA	SUPV3L1 encodes a helicase that is localized in the mitochondria. It has been shown in vitro to possess both double-stranded RNA and DNA unwinding activity that is ATP-dependent	Gene Matched (understudy). One study was published recently with no further confirmation^89^
10BN7500	13 Years	Male	Yes	Intellectual disability	TACC2	TACC2(NM_206862.4):c.8260G > A (p.Ala2754Thr)	homozygous	Yes	rs538968585	0.00002	0/4	TACC2 is involved in the microtubule-dependent coupling of the nucleus and the centrosome and in the processes that regulate centrosome-mediated interkinetic nuclear migration (INM) of neural progenitors^90^. TACC2 is a tumor suppressor and an oncogenic protein^91^	No published genotype–phenotype association
10SS8000	7 Years	Male	Yes	Moderate to severe intellectual deficiency, behavioral abnormalities, facial dysmorphism, and ophthalmological abnormalities	THUMPD1	THUMPD1(NM_017736.5):c.774_776del (p.Leu258del)	homozygous	Yes	rs772419789	3.98E-06	NA	THUMPD1 is highly expressed in the brain and it is a unique adaptor protein that regulates tRNA acetylation interacts through interaction with NAT10^92^	Published^93^
10DK15900	2 Months	Female	Yes	Variable signs of cardiomyopathy, neonatal pulmonary hypertension, hypophosphatemia, chronic lung disease, developmental delay, and brain basal ganglia calcification	XPR1	XPR1(NM_004736.4):c.1811G > A (p.Arg604Gln)	homozygous	Yes	NA	Absent	4/4	The XPR1 protein mediates phosphate export from the cell and binds inositol hexakisphosphate and related inositol polyphosphates, key intracellular signaling molecules. Mutations in XPR1 gene are associated with the dominant condition of idiopathic basal ganglia calcification-6^22,23^. No ID phenotype in this condition	The patients' phenotype is strikingly different from the reported phentyope, with no past ID phenotype described. No biallelic variants for XPR1 were described
10MS4500	4 Years	Female	Yes	Variable signs of cardiomyopathy, neonatal pulmonary hypertension, hypophosphatemia, chronic lung disease, developmental delay, and brain basal ganglia calcification	XPR1	XPR1(NM_004736.4):c.1811G > A (p.Arg604Gln)	homozygous	Yes	NA	Absent	4/4		
50DK4600	2 Years	Male	Yes	Variable signs of cardiomyopathy, neonatal pulmonary hypertension, hypophosphatemia, chronic lung disease, developmental delay, and brain basal ganglia calcification	XPR1	XPR1(NM_004736.4):c.1811G > A (p.Arg604Gln)	homozygous	Yes	NA	Absent	4/4		
63MS1200	3 Years	Male	Yes	Variable signs of cardiomyopathy, neonatal pulmonary hypertension, hypophosphatemia, chronic lung disease, developmental delay, and brain basal ganglia calcification	XPR1	XPR1(NM_004736.4):c.1811G > A (p.Arg604Gln)	homozygous	Yes	NA	Absent	4/4		
22SN9300	6 Years	Female	Yes	Seizures and neurodegenerative disorder (? Leukodystrophy) in addition to also cystic fibrosis	ZFYVE28	ZFYVE28(NM_020972.3):c.2015_2016del (p.Ser672CysfsTer67)	homozygous	Yes	NA	Absent	NA	ZFYVE28 has been found to operate as a negative regulator of epidermal growth factor receptor (EGFR) signaling. It promotes EGFR degradation in endosomes when not monoubiquitinated, thereby terminating EGFR signaling. The highest expression is seen in the brain at the cortex and cerebellum. These patients also have cystic fibrosis phenotype explained by CFTR pathogenic variants	No published genotype–phenotype association

*MetaLR, MetaSVM, MetaRNN, REVEL.

**Table 2:** Pitfalls and challenges of exome analysis.

Pitfalls of WES	Examples
Sequence related	Unmapped genome sequence, PCR amplification artifacts or capture target Structural variants include complex or Copy Number Variants Mitochondrial genome or epigenetic variants Mosaic or uniparental disomy variants Variants in GC rich regions, pseudogenes, or repetitive and homologous sequences
Annotation and prioritization	None-canonical (Alternative) transcripts, mini exons or gene definitions OMIM-database entries delay or lag Splicing and intronic variants Synonymous that affect splicing enhancer, suppressor or induce cryptic splicing site High allelic frequency in a population but pathogenic
Clinical and phenotype	Strikingly different phenotypes or different modes of inheritance from what was described previously Two or more genetic conditions within the same family Variability of the phenotypes Incomplete clinical reevaluation after negative exomes

## Conclusion

In conclusion, using WES to identify the novel causes of human disease has changed the research landscape of genetic and neurodevelopmental disorders. Although WES is comprehensive technology, its limitations must be considered when negative results are obtained. The pitfalls of WES can potentially reduce the effectiveness of this technique in biological and medical research as well as in clinical settings. Finally, it is worth emphasizing that identifying a likely candidate gene is often just the start of a long process to confirm the variant’s pathogenicity.

## Supplementary Information

Supplementary Information 1.

Supplementary Information 2.

## Data Availability

The authors confirm that the data supporting the findings of this study are available within the article and Supplementary material. Further derived data are available from the corresponding author upon reasonable request. The datasets generated and/or analysed during the current study are available in the [Clinvar] repository, [https://www.ncbi.nlm.nih.gov/clinvar/; submission number  SCV002574702 to SCV002574743].
