# Supplementary material for: The diagnostic yield, candidate genes, and pitfalls for a genetic study of intellectual disability in 118 middle eastern families
Source: Sci Rep. 2022 Nov 7;12:18862. doi: 10.1038/s41598-022-22036-z (PMC9640568; doi:10.1038/s41598-022-22036-z)
Supplement: Supplementary file 1 — Supplementary Information 1. [file 41598_2022_22036_MOESM1_ESM.docx]

# **SUPP S1: MATERIALS AND METHODS**

#

# **Subjects**

Affected individuals enrolled in this study were selected from patients who attended Sultan Qaboos University Hospital or the National Genetic Center at Royal Hospital, Muscat, Oman. Medical geneticists were engaged in selecting the families for the study and characterizing the clinical phenotype. The target patients included in this study presented with global developmental delay or intellectual disability. The assessment included clinical developmental milestones screening tests. When feasible objective assessments including Vineland Adaptive Behavior Scales (Third Edition) or Stanford–Binet Intelligence Scales, Fifth Edition for older children were completed. Severe phenotypes causing death within the neonatal or early infantile period were also included as far as a neurological phenotype is evident, for example, seizures, hypotonia, or brain malformations.

**Inclusion criteria included:**

1. Global developmental delay/intellectual disability phenotype
2. Patients with negative genetics investigations are performed based on clinical suspicion including targeted genetic testing (known genetic condition or familial mutation), genetic panels, Clinical Exome Sequencing (CES), Fragile-X, or chromosomal microarray (chromosomal deletion/duplication studies).
3. Families with a strong autosomal recessive pattern of inheritance and/or consanguineous were selected. Prioritization was given to families with multiple affected individuals.

**Exclusion Criteria:** previously known molecular diagnosis.

The diagnostic pathway for these patients included a full assessment by a medical geneticist, performing investigations and diagnostic testing as indicated by the clinical judgment and differential diagnosis, including metabolic and target genetic testing. Fifty-two affected individuals underwent clinical exome (years 2013-2015). Chromosomal microarray was completed on a third of the cohort, and fragile X was only done on males with possible X-linked inheritance (less than 15). At least 40 affected individuals were genetic testing naïve because, at as at the beginning of the study, clinical genetic testing was unavailable for all patients.

# **Ethical approval**

The Medical Research Ethical Committee approved the study of Sultan Qaboos University (SQU MREC#1362). Written informed consent was obtained from all participants or their guardians. Genetic counseling was provided to all families, which explained expected research findings, including any diagnosis, general genetic concepts, or incidental findings. Medical geneticists were responsible for delivering the results back to the families.

# **Genomic Analysis**

# **DNA extraction**

Peripheral EDTA blood was collected to obtain DNA samples as well as other tissue specimens, such as saliva, or cheek swabs from parents, affected individuals, and healthy siblings when required. When samples were available from a previously stored DNA in the clinical laboratory, an aliquot was taken after obtaining written consent from the families. Otherwise, to obtain DNA samples, blood samples were collected in sterile air-vacuumed tubes containing ethylene diamine tetra acetic acid (EDTA) from all participants. Following the manufacturer’s protocol, the Qiagen DNA maxi kit (Qiagen, Germany), was used for DNA extraction.

**Whole-Exome Sequencing**

Whole exome sequencing (WES) was performed to identify the genetic variants that might be causative of the disease in the affected participants. Exome sequencing was outsourced to a clinical-grade service laboratory for rapid results and cost-effectiveness. In brief, DNA was barcoded and enriched for the coding exons of targeted genes using hybrid capture technology (Agilent SureSelect Human All-exons-V6 or V7). Prepared DNA libraries were then sequenced using a Next-Generation Sequencing (NGS) Illumina technology [Hiseq2500, Hiseq4000, or NovaSeq6000] of 150 bp paired-end, at 150-200X coverage. The reads were mapped against UCSC GRCh37/hg19 or GRCh38/hg38 by Burrows-Wheeler Aligner (BWA 0.7.12). Genome Analysis Tool Kit (GATK 3.4 or Haplotypecaller in GATKv4) was used for variant calling.

Raw data were analyzed using in-house annotation and analysis pipelines. Collected raw data (BAM and VCF files) were pre-processed using BCFtools (v.1.5), and then annotation was completed using ANNOVAR tools (version release 2015 Dec14 with its updates). Left alignment and decomposition of multiallelic sites were completed for all raw files using the same pipeline. Variant filtration was conducted only to keep novel or rare variants (≤ 1%). Publicly available variant databases (1000 Genomes, Exome Variant Server, and GnomAD) were used to determine the frequency. Also, an in-house database of 1564 exomes was used to filter out common or benign variants specific to our population.

Filtration and prioritization were conducted as follows: only rare coding or splicing variants were considered initially. The phenotype and mode of inheritance were considered. The following criteria were then used to prioritize variants; high impact or highly damaging missense, a CADD score ≥ 15, and a variant shared between the affected individuals. Sanger sequencing was done to confirm variant segregation within family members. Supporting evidence for candidate genes include population frequency supports, genic intolerance to such types of variants or genes knock-out, and in-silico predictions tools indicating damaging effect. Accurate deleteriousness prediction for nonsynonymous variants is essential to infer the pathogenicity of the prioritized variants. Although many deleteriousness in-silico prediction methods have been developed, the results are inconsistent. In our study, we used tools that integrate and combine scores from multiple individual prediction methods. We selected the known tools named MetaLR, MetaSVM, MetaRNN and REVEL. These tools integrate data from over 20 prediction scores, for example, SIFT, PolyPhen-2, GERP++, MutationTaster, Mutation Assessor, FATHMM, LRT, SiPhy and PhyloP. In addition to the allele frequency information from the 1000 Genomes Project (1000GP), ExAC, and gnomAD. In our analysis, these tools were used as part of the evidence for pathogenicity in combination with evidence from the phenotypic and variant characteristics. Also, data for gene function and network, gene expression, and animal models were considered.

For cases where an answer was not obvious or a candidate gene was selected, we applied copy number variant analysis using ExomDepth. ExomeDepth_1.1.15 R library was used to call the CNVs. First, the count data were created from BAM files using the “getBamCounts” from the ExomeDepth library, creating a variable of BAM files that includes the test BAM plus 5 aggregate references built an optimized reference set using the “select.reference.set formula”. The CNV calling step applies the beta-binomial model using the “new” function and the “CALLCNVs” function in the ExomeDepth Library. The CNV calls are further annotated with “Conrad.hg19”, which identifies the overlap with a set of common CNVs and “exons.hg19” that specifies exon positions for the hg19 build of the human genome, both Conrad.hg19 and exons.hg19 are available within the ExomeDepth library.

The ACMG guidelines on variant interpretation and classification were followed. Variants were classified into three groups. Disease-causing variants are pathogenic or likely pathogenic variants in known disease-causing genes associated with the reported phenotypes of the affected patients. Possible disease-causing variants are rare and damaging variants but of uncertain significance (VUS); seen in known disease-causing genes that overlap with the patient the phenotype. The third group was variants in candidate genes, which were predicted to be deleterious and found in genes that were not confirmed previously to be implicated in human disease.

# **Mutation Analyses**

Mutation analysis was performed to establish the causative role of the prioritized variants. In most of the recruited families, exome sequencing was done in a proband and affected siblings. Identified prioritized variants needed to be validated and segregation analysis completed in multiple family members using Sanger sequencing. Sequence validation and testing for each candidate variant segregation within the remainder of the family members were done through conventional Sanger sequencing following standard procedures.
